# Critical Coping Challenges Facing Caregivers of Persons Living with HIV/AIDS and other Terminally III Persons: The Case of Kanye Care Program, Botswana

**DOI:** 10.4103/0973-1075.58456

**Published:** 2009

**Authors:** Simon Kangethe

**Affiliations:** Gaborone City, Botswana

**Keywords:** Care giving/caregivers, Community home based care program, Coping challenges, HIV/AIDS

## Abstract

**Aim:**

This article aims to identify and explore the needs, gaps, and coping challenges faced by the Kanye CHBC caregivers.

**Objective:**

To provide the Kanye primary caregivers with an opportunity to explore, discuss, and brainstorm the care giving challenges that impede their coping process.

**Materials and Methods:**

The study was exploratory in nature and attracted qualitative design. Eighty-two primary caregivers aged between 18 and 85 years were involved in 10 focus group discussions steered by an interview guide; while five CHBC nurses were subjected to one-to-one in-depth interviews still guided by an interview guide that differed only slightly with the one for the caregivers.

**Results:**

The following aspects were found detrimental and affecting the productivity and coping capacities of the primary caregivers inadequate counseling and debriefings; lack of motivation and incentives; inadequate supervision visits; and lack of support groups to facilitate information sharing and encouraging one another.

**Conclusion:**

This article recommends counseling and debriefings; formation of caregivers support groups; motivation strategies to be put in place; and provisions of adequate care package and food basket.

## INTRODUCTION

It was the researcher's interest to assess the operational challenges in the Kanye CHBC program and find out how much the program was meeting the Botswana CHBC program objectives as spelt out in National AIDS Control Programme (NACP 31).[1] The CHBC[2] policy provision saw the institutionalization of CHBC program and its integration into the mainstream health facilities. The researcher was prompted to assess the challenges because of the anecdotal complaints from the caregivers and the community at large about the program operations. The findings, he hoped, would be a platform of advocacy to make positive changes.

Since some researchers in Botswana had pointed to the program's unmet operational challenges,[3,4] the researcher was interested in assessing how much of these challenges had been met or whether the situation was getting out of hands. During the time of research (2005-2006), for instance, HIV/AIDS prevalence was still unacceptably high, with national sentinel survey recording more than 30%, while the program was allegedly experiencing higher death toll.[5] These are indicators of coping challenges among the caregivers. It was therefore imperative that the whole operations of the program challenges, gaps, and dilemmas be informed by research through tapping the caregivers' views, attitudes, thinking, and beliefs systems.

HIV/AIDS has placed undue burden to the caregivers who take care of the people living with HIV/AIDS.[6,7] These caregivers suffer neglect in that while the government has splendid policies to cover people living with HIV/AIDS, the caregivers' policies have not been well developed let alone operationalized.[8]

Immense support system to the caregivers is, therefore, necessary if the level, efficiency, and effectiveness of the care giving in Botswana are to change the face. Poverty of the caregivers has been the biggest challenge besetting the process.[4,9] Serious poverty mitigation factors need to be put in place to salvage the dwindling work environment of the caregivers and to make coping manageable. The working environment has been riddled with immense challenges that results to workload fatigue and burnout.[10,11] This has made coping difficult. Research to bring to the fore these challenges is important as it brings hope that measures and strategies could be put in place to redress the challenges and make coping easier. The aim of this article is to explore the operational gaps and challenges in the care giving process that make coping of the caregivers a daunting task.

## MATERIALS AND METHODS

### Research design

Qualitative design was used in the study. Qualitative research is concerned with meaning and sense that the respondents comprehend from the phenomenon under study. To clearly understand the meaning and sense that the respondents own calls for the exploratory skills, such as probing embedded in the qualitative design. Participant observation to capture the mood and temperaments of the caregivers forms an important tool for qualitative design. The researcher's view and his conceptualization of phenomenon interact with the respondents to shape the final reality of the respondents.[12]

### Research instruments

Two slightly different interview guides were used—one to guide 10 focus group discussions (FGDs) with the caregiver respondents and the other to administer the nurses with one-to-one interviews. The guide had the profile section where the respondents' personal details such as age and gender were recorded. The rest of the questions were open-ended in that their role was only to stimulate and generate discussions to make the caregivers spill out the reality of events in the care-giving environment. The results of a pilot study involving only five caregivers and one nurse had served to cleanse the instrument of its ambiguities.

### Sample selection criteria and procedure

All the 140 registered primary caregivers as they appeared in the CHBC register were conveniently picked for study inclusion into 10 focus group discussions. Eighty-two (59%) registered primary caregivers turned up for focus group discussions. Five nurses who supervised the caregivers were subjected to one-to-one interview. All the FGD sessions lasted between 60 and 90 min. The CHBC register in each clinic helped to identify the number of the primary caregivers served by each clinic and hence made the sample grouping for the FGD easier.

FGDs—facilitated by the researcher and two research assistants—collected data on occupational challenges, gaps, and dilemmas that the caregivers were going through. While one research assistant handled the audio microphone to the discussant on the floor, the other was making notes and the rest of the team noted the respondents' body gestures, temperaments, and tones. The primary caregiver, therefore, formed the unit of analysis.[12]

### Ethical and legal requirements

To ensure that the study was politically correct, all the legal and ethical channels and protocols had been observed. The researcher through the clinic heads had met the respondents well in advance, discussed the study objectives, the research process and its importance to them, and their region and the country at large. The researcher had then issued consent forms that were signed by those who were in agreement with the research process. In the forms, the researcher had promised and committed to treat the respondents with all the dignity they deserved and to maintain confidentiality and anonymity. Respondents were informed of their right and freedom to withdraw voluntarily if they wished to do so or if they felt uncomfortable with research proceedings.[12] The researcher had applied for a license, a written sample of a proposal which had to pass the test of the Human Research and Development Committee (HRDC) Board of the Ministry of Health. He had been issued a license upon meeting all the application conditions. The researcher had then to write a letter to the Southern District Council asking for authority to enter into the field to collect data. The letter was attended to by the Southern District Council (SDC) matron, who wrote to all the clinic heads asking them to assist the researcher in his data collection exercise.

### Data analysis, interpretation, and bias reduction

The data analysis process started with putting together stacks and stacks of crude data that had been audio-taped and transcribed. The data were sorted by the use of codes. This made the data to be arranged easily in groupings that gave rise to themes and subthemes. Quotes, words, analogies, proverbs, and jotted notes were used to inform data collection, while tables and graphs were used to present the data and the findings.

To reduce data and result bias, there was double translation of the instruments, that is, translation from English to Setswana and then from Setswana to English by two independent translators, the two parties coming together to settle on the difference. To strengthen data reliability and validity, the two interview guides or the instruments used only differed slightly and the two sets of responses confirmed and cross-checked each other.

### Research domain

The data for this article were obtained from empirical research done in December 2005 and January 2006 at Kanye village. The village, one of the oldest in the country, had a population of over 40,000.[13] It is well endowed with five clinics and two health posts and a bigger Seventh Day Adventist (SDA) referral hospital. Although Kanye CHBC was one of those that were doing well by government standards, it was experiencing a high death toll among the CHBC clients. However, Southern District HIV prevalence rate was 25.7% according to 2003 national HIV/AIDS statistics.[14]

## THEORETICAL FRAMEWORK

### Coping theories

Under normal and natural phenomenon, coping constitutes the actions taken by individuals and animals when faced with stressful events in order to lessen the threat to them. Stress is a state of tension felt in the presence of an object or a task that is perceived as presenting a challenge to one's safety or self-esteem.[15] Stress emanates when there is a perceived discrepancy between environmental demands and one's ability to meet those demands. Stress has both psychological and physiological causes and effects. For an individual to continue functioning in an adaptive way, he/she must learn to cope with stress.

There are many ways to cope, varying from avoiding stress or denial of stress at one extreme to seeking and confronting the source of stress. According to Magill,[16] coping attempts either to reduce the demand, to reduce its effects, or to help one change the way one thinks about the demand. It also attempts to eliminate or moderate the initial source of the stress reaction (stimulus-directed coping), reduce the magnitude of the stress response (response-directed coping), or change the way the stressor is perceived (cognitive coping). For individuals such as caregivers in the HIV and AIDS field, both the internal factors (such as knowledge) and external (such as money or friends) are necessary to help one cope with a stressful event. Social support or resources provided by other people to enhance one's self-esteem, psychosocial support, and assistance are critical in helping the coping process.[7,16]

## FINDINGS

Age was found to be a crucial factor affecting care giving with 46 caregivers (56%) being 50 years and above and 28 caregivers (34%) being 60 years and above. The study revealed that most caregivers being women and especially those above 60 years were physically not strong enough to stand the care-giving demands, making coping difficult. On literacy level, 29 (35%) caregivers had never been to school, 32 (39%) had only primary education, while 17 (21%) had secondary education. Only 5% of the caregivers had tertiary education. Illiteracy was found to have negative influence on care-giving quality and making coping process difficult. This is because most of the low-literate caregivers (who were also elderly) had problems of accessing care-giving educations, following the medical and hygiene protocol, and following disease progression of their clients.

On economic front, many caregivers decried poverty. Seventy-two (88%) caregivers did not have any income to support themselves. On gender, data indicate that the program faces serious skewed gender dimension with 80 (98%) being women and only two (2%) men.

### Poverty of the caregivers and inadequate nutrition

About three-quarters of the caregivers indicated that care giving experiences, difficulties, and dilemmas occurred because of the fact that most of them were poor. Seventy two caregivers (88%) had no economic engagement to make a living, while only 10 (12%) were occupied with some marginal economic activities. The most common problem quoted by almost all the respondents in different focus group discussions was lack of adequate and necessary nutrition for their clients. Caregivers indicated that the food basket offered to caregivers for their clients was inadequate and, therefore, not meeting their nutritional needs adequately. This posed serious implications and challenges on the health of some clients especially those on antiretroviral (ARV) drugs who needed special diets. This forced the caregiver to look for supplementary feed, which may not be available. This was made worse by the fact that that the social workers' assessment results for the food basket were taking too long to be released. Even though not all the caregivers got the food basket, those who were getting it complained that the food was inadequate and standard for all the clients. This standardization is not fair given the fact that different patients have different food requirements due to varying levels of ailments.

Because of poverty, caregivers conceded they were not able to provide what their clients desired. The following sentiments were echoed by many caregivers in all the 10 focus groups

“The patient does not understand. You give him/her the food you have, she/he refuses, yet you have no money to buy her/him what he/she wants as you are unemployed.”

“*We are poor and have nothing else to do except care giving.”*

“Food is inadequate and does not meet the needs of the clients.”

“This food is not adequate to the client. The food basket is also standardized for all the clients, yet the ailments are different”

“some food items are not there in the food basket supplier shop. Our sick clients and ourselves are suffering. We need help.”

“The client wants good food, you give him/her thick sorghum porridge (one of the common staple food for an ordinary Motswana); s/he vomits.”

“We are sometimes given stale and expired food items by shop owners.”

“Not all the sick clients get the food basket. We do not understand why because most of our clients need food assistance.”

“Some of us have been waiting for the assessment results of the social workers for a long time in vain.”

### Weak community support system

Empirical findings indicated that caregivers are inadequately assisted by relatives, friends, neighbors, private individuals, grassroots traditional and political leaders, and other service delivery networks like the NGOs and CBOs. The sentiment below was unanimously echoed by majority of the caregivers:

“Relatives and family members help here and there, but very little.”

This indicates possibilities that caregivers could get overwhelmed making coping process difficult. It is also an indication of the possibility of family members and relatives neglecting or leaving care giving in the hands of the caregiver alone. Demonstrating the pain and agony a caregiver undergoes while left to carry out care giving alone, an 18-year-old female caregiver broke into tears explaining how all her brothers and sisters left her alone to take care of her ailing father.

“I cannot get time to go and look for a job as I am left alone at home caring for my father. They all (brothers and sisters) went for good leaving, I am struggling with care giving.”

### Inadequate supervision and counseling by healthcare personnel

Seventy (85%) Kanye caregivers indicated disappointments as far as visiting, counseling, and supervision from the healthcare personnel such as nurses, counselors, social workers, and doctors were concerned. They indicated that there were only a few cases when nurses, especially community home-based nurses, checked, counseled, or visited the caregivers and their clients. This heralds lack of care guidance, direction, motivation, and quality of care giving. This has a negative bearing toward care giving productivity and coping in general. The following sentiments were expressed by caregivers:

“The health personnel do not come to supervise us.”

“The health personnel only counsel and visit a few people. We use our natural instincts to do care giving.”

“We do not see the health personnel coming to encourage us. We rarely get their help.”

“We do not get counselors to offer counseling in our caring duties.”

Caregivers reported that they preferred health workers to visit the households in order to supervise them, counsel them, and to encourage the patients to take their drugs. Such visits would improve the contribution to care giving and enhance their coping process.

### Inadequate support from the social workers

Virtually all the respondents were dissatisfied with the social workers' services. Because of poor service delivery from social workers, it took too long for the clients who are assessed for a food basket to get their assessment results and, therefore, the food basket. This punished the caregiver who is responsible for the welfare and nutritional requirements of the client. The following sentiments that run across various FGD bear testimony:

“The social workers do not perform their duty effectively. They do not help us.”

The respondents associated the inadequacies of the food basket to the ineffectiveness of the social workers. This is because social workers took too long to process the assessments for food basket to an extent that some clients die while waiting for the results.

### Inadequate motivation and incentives

Forty-one caregivers (50%) indicated that Kanye caregivers are not exposed to any motivation in their care-giving work. The program was demotivating, demoralizing, lacking in incentives, recognition, rewards, or any strategy to motivate the caregivers. This had the effect of making coping immensely challenging. Lack of incentives, the respondents reported, was the reason why care giving does not attract young women and men. Giving incentives, according to the respondents, could include giving stipends and rewards, bonuses, encouraging words, food packages, toiletries, visits and supervision, relieving somebody before s/he gets overwhelmed, allowing caregivers work in turns, and monetary payments. The respondents pinned lack of psychosocial support like counseling, social support from relatives and community in general, and inadequate care package as factors responsible for poor motivation and reduced care productivity. These factors made coping very challenging: They echoed the following sentiments:

“Incentives are not there to encourage us.”

“If you want men and young women to participate in care giving, give them incentives.”

## DISCUSSION

Kanye caregivers were found to be of low economic status, low education, and the programs immensely suffered skewed gender dimension. In a study by Kang'ethe[17] in Kanye, more than 50% of the caregivers were either illiterate or semi-illiterate. On gender skewedness, another study carried out in Botswana by Munodawafa[18] had all but one female caregiver in Tutume, while in Molepolole, all caregivers were women. This is an indicator of women being left solely to handle care-giving tasks to either float or sink. This has immensely compromised their coping process. This, according to feminists such as Finch and Grove,[19] constitutes exploitation of the female gender.

Recommendation

- Strategies to woo the relatively younger and educated persons into the CHBC program need to be explored. This could complement and help alleviate the illiteracy of the caregivers. Strong advocacy and education challenging men and the young to fully participate and complement the duties of the elderly caregivers need be scaled up. The role of leadership at all levels to take a leading role is critical.

The poverty of the caregivers in Kanye CHBC program finds support from other similar studies. In a study by Mojapelo *et al.*[4] in Botswana, 85% of the caregivers were unemployed and quoted poverty and inadequate food as the biggest stumbling block besetting care giving. Similar studies in Kweneng by Khan and Stegling[9] found glaring poverty among the caregivers as evidenced in lack of most basic necessities, with lack of food being the gravest. Inadequate food poses immense challenges in this era of HIV/AIDS because of the fear that ARV drugs may not work well. The fact that the program had very high death toll also posed a great concern.[5] According to South Africa Development Cooperation (SADC) Executive Secretary, Dr. Salmao,[20] the ARV rolls out may not achieve desired results if food security is not adequately addressed.

Recommendation

- The Government and other care managers need to scrutinize and explore further the issue of the food basket to ARV clients' needs. The food basket needs to conform to the clients' nutritional needs if administration of the ARVs and CHBC programs are to succeed.
- The Government in collaboration with care managers needs to ensure that the food basket is individualized and disease specific, well balanced, and adequate.
- Strategies and mechanism of funding the caregivers to start small and viable income-generating projects could possibly address the poverty and meet the food needs of the caregivers.[21] Inadequate support of the caregivers by communities presents a worrying state of affairs. This appears to be a departure from past practice when research proved that Africans have always been cooperative and helpful to their distressed brothers and sisters.[22] Studies done in Kweneng in Botswana on care giving by Khan and Stegling[9] found that caregivers felt unsupported by their families, relatives, and community at large, while findings from Chirumhanzi CHBC program in Zimambwe has not had a great deal of support from the local chiefs or village health workers. Some chiefs and some health workers feel they should be paid for their involvement.

On the contrary in other countries, NGOs and other civil society bodies are very active in community activities, care giving being one of them. The *Sanpatong* CHBC program in Thailand has identified support and acknowledgement from neighbors and community as one of the special needs of care giving. Many organizations like *Thai Red Cross Society* have led numerous fundraising to help the activities of the care programs.[23] In Brazil, *Hope Project,* a community care project, benefits greatly from community support. This is expressed in contributions of materials or services. Fundraising or recreational events often receive support from local businesses.[23]

Kang'ethe[8] contends that the unfortunate dwindling of the black African gregarious spirit with time is a result of black Africans embracing and favoring modernization, civilization, westernization, and Eurocentric values of individualism—and abandoning the traditional communal or socialistic style of living that saw people doing work together and helping one another for the good of all in the society.[22] Joy Phumaphi, the 1999-2002 Botswana Health Minister, encouraged Batswana to go back to the roots of human coexistence and embrace values of helping one another and assist care programs economically and psychosocially when she said, “home-based care is taking us back to the roots of human coexistence… by having responsibility to one another. If we hold hands through this tragedy we will be able to retain our humanity and we will come out of this epidemic as a stronger community.”[24] The current call by Botswana Government for communities to cherish and work to fulfill Botswana's vision 2016 through its pillars like “being a just, caring, and compassionate society” is a call for people to value and inculcate the spirit of helping one another.[25]

Recommendation

- The researcher recommends a paradigm shift of attitudes, ideas, thinking among community members, and their leaders to support care giving if the program is to succeed. The government should also increase community assistance as the CHBC program is a collaborative effort between the Government and the community.
- Campaign advocacy targeting all the civil society bodies to increase and step up their assistance to community care programs should be scaled up. National and local campaign advocates, lobbyists, and leaders, both at local and national levels, should be at the forefront to challenge all to increase their community support to care giving.

A study by Atta and Fidzani[2] in Botswana indicated how caregivers longed to be visited, supervised, encouraged, and be given direction on care giving. Care giving, therefore, being psychologically draining and burdensome, calls for remedial services to caregivers to help invigorate and replenish their energy in order to maintain their state of well-being.[6,7,21] In their Kweneng study, Jacques and Stegling[3] found that healthcare providers were failing to supervise caregivers in their care-taking, while studies by Atta and Fidzani[2] indicated that 95% of the caregivers lacked supervision support from health workers. In 2002, the Government through Joy Phumaphi (former Health Minister) complained on the issue of the family welfare educators (FWE) who stayed in the clinics instead of helping the clients at their homes.[26] This is an indication of the Government's acknowledgment that psychosocial and psychological support to caregivers is lacking. This could possibly explain the state of low productivity and quality of care discharged by caregivers in Botswana Kanye caregivers not withstanding. The phenomenon could also be responsible for poor coping process among the caregivers.

Nurses Association of Botswana[7] emphasizes the importance of counseling in facilitating the grieving process by being there; by nonjudgmentally listening and assuring the clients that they are not going crazy; and that the acute pain they are experiencing is grief in process and the state will not last forever. Grieving process experienced in care giving can temporarily be disabling and working through it by counseling ultimately brings strength.[27] Counseling, therefore, is a very important input especially in this era of AIDS. The caregivers and their clients need to be empowered both psychologically and psychosocially. Counseling helps to make a caregiver come into grips with the reality of the problem situation and make one feel s/he is not going crazy, instills hope and confidence, make one free to seek support, and share with others with the result of reducing the psychological and emotional burden.[6,7,21]

Recommendation

- It is recommended that the government, NGOs, and other care-friendly organizations put in place a strategy ensuring and forcing caregivers' supervision, counseling, and monitoring of the care programs. This would improve the coping challenges experienced by the caregivers. This is likely to reduce burn out of the caregivers and make coping of the caregivers an easier task.

The role of social workers in the care programs is to make counseling visits to the caregivers for psychological empowerment and assess their socioeconomic conditions for possible help intervention; facilitate the process of positive change of attitudes and norms relating to care giving among the caregivers; and therefore give way to increased care-giving productivity. The researcher, having been in the study area, understood the staffing problem the department of Social and Community development (S and CD) was experiencing, with social workers suffering from “overload burnout.”

Recommendation

1. It is recommended that the government employs adequate social workers in the community care programs if the care programs were to succeed in psychosocially empowering the stakeholders of the care programs. The NGOs and other care programs should also be visible in complementing government effort by deploying social workers and other necessary staff to assist in issues of care giving.
  Kanye caregivers lacked incentives and motivation. The importance of providing caregivers with incentives has been emphasized and addressed in other countries. In Namibia, for example, caregivers are given bonuses and a little money to support their living, while TASO (The Aids Service Organization) caregivers in Uganda are paid more than other Government workers of their caliber. The organization proposes to introduce a policy of assisting at least two children of a sick caregiver with school fees. In Mozambique, the government has made a policy to pay the caregivers an allowance that is 60% of the government minimum wages.[28]
2. It is recommended that the government, NGOs, and care authorities create a provision or an environment resulting in caregivers' recognition and appreciation of their tasks. Considering some monetary allowance would be important. This would be an important factor that can raise the caregivers' morale.

## CONCLUSIONS AND RECOMMENDATIONS

The government and care managers need to go to the drawing board and look at the policy and operationalization of care programs. Immense goodwill is necessary to analyze all the challenges and put in place redressing strategies. Putting in place a mechanism of rewards, recognition, and incentives to raise the morale and motivation of the caregivers is especially critical.
